# Isolation of All *CD44* Transcripts in Human Epidermis and Regulation of Their Expression by Various Agents

**DOI:** 10.1371/journal.pone.0160952

**Published:** 2016-08-09

**Authors:** Kwesi Teye, Sanae Numata, Norito Ishii, Rafal P. Krol, Atsunari Tsuchisaka, Takahiro Hamada, Hiroshi Koga, Tadashi Karashima, Chika Ohata, Daisuke Tsuruta, Hideyuki Saya, Marek Haftek, Takashi Hashimoto

**Affiliations:** 1 Department of Dermatology, Kurume University School of Medicine, and Kurume University Institute of Cutaneous Cell Biology, Kurume, Fukuoka, 830–0011, Japan; 2 Department of Dermatology, Osaka City University Graduate School of Medicine, Osaka, Japan; 3 Division of Gene Regulation, Institute for Advanced Medical Research, Keio University School of Medicine, Tokyo, Japan; 4 University of Lyon 1, EA 4169 and CNRS, Lyon, France; NYU Langone Medical Center, UNITED STATES

## Abstract

CD44, a cell surface proteoglycan, is involved in many biological events. *CD44* transcripts undergo complex alternative splicing, resulting in many functionally distinct isoforms. To date, however, the nature of these isoforms in human epidermis has not been adequately determined. In this study, we isolated all *CD44* transcripts from normal human epidermis, and studied how their expressions are regulated. By RT-PCR, we found that a number of different *CD44* transcripts were expressed in human epidermis, and we obtained all these transcripts from DNA bands in agarose and acrylamide gels by cloning. Detailed sequence analysis revealed 18 *CD44* transcripts, 3 of which were novel. Next, we examined effects of 10 different agents on the expression of *CD44* transcripts in cultured human keratinocytes, and found that several agents, particularly epidermal growth factor, hydrogen peroxide, phorbol 12-myristate 13-acetate, retinoic acid, calcium and fetal calf serum differently regulated their expressions in various patterns. Furthermore, normal and malignant keratinocytes were found to produce different *CD44* transcripts upon serum stimulation and subsequent starvation, suggesting that specific CD44 isoforms are involved in tumorigenesis via different CD44-mediated biological pathways.

## Introduction

CD44 is a cell surface proteoglycan implicated in multiple cell functions, including adhesion, migration, activation, recirculation and homing of lymphocytes, hematopoiesis, tight-junction assembly and tumor metastasis [1–4]. Recent studies suggested that CD44 is a cancer stem cell marker [5,6]. CD44 is recognized as the principal receptor for hyaluronic acid (HA) [7] and CD44-HA interactions have been demonstrated to affect many physiological and disease processes including promoting keratinocyte activity, improving abnormal epidermal function and melanoma and prostate cancer development [8–11].

*CD44* gene locates on the short arm of chromosome 11 and is composed of 19 exons [12–14] (S1 Fig). Exon 18 is noncoding. Transcripts of *CD44* undergo complex alternative splicing of at least 9 of the 18 coding exons, resulting in many functionally distinct isoforms [14]. The smallest CD44 molecule is called standard CD44 (CD44s). Larger variable isoforms, such as CD44v6, CD44v9, CD44v2-10 and CD44v3-10, are generated by insertion of single or multiple alternative exons into a single site of CD44s [14] (S1 Fig). Alternative splicing of *CD44* would theoretically result in more than 800 transcripts [15]. However, only a part of transcripts are expressed in a tissue-specific and context-dependent manner [3,16,17]. It is well documented that particular CD44 variants are involved in pathophysiology of several malignant tumors [18–21]. CD44 expression [22,23] and alternative splicing [24] were also implicated in tumor metastasis and epithelial-to-mesenchymal transition of cancer stem cells [25].

CD44 functional diversity is further complicated by post-translational modifications such as phosphorylation and glycosylation. Most alternatively spliced exons produce peptides with different N- or O-linked glycosylation sites, thus giving increased or decreased functionality to a particular CD44 variant [16]. In addition, the presence of heparan sulfate or chondroitin/dermatan sulfate glycosaminoglycan (GAG) side chains on CD44 molecules has important functional consequences [3].

Since different tissues express different *CD44* variants with various exon combinations, it is important to know exactly which CD44 variants are expressed in normal and diseased states. To date, however, the characteristics and pathological roles of different CD44 variants have not been extensively studied in most tissues including skin. Determination of the combination of alternative forms of *CD44* and their transcripts in tissues is hampered by varying expression levels of transcripts with different sizes. In this study, we devised a cloning strategy that enabled us to isolate and designate all *CD44* transcript variants present in human epidermis. We also investigated the effect of various agents on the expression of *CD44* transcript variants in cultured human keratinocytes, in order to shed more light on specific biological pathways mediated by particular CD44 isoforms in human skin.

## Materials and Methods

The studies were approved by the internal review board of Kurume University School of medicine.

### Cell lines and primary cells

Chinese hamster ovary (CHO) K1, epithelial-like cells were obtained from RIKEN Bioresource center (Tsukuba, Ibaraki, Japan). CHO-K1 cells were cultured in Ham F12 medium supplemented with 10% fetal calf serum (FCS). Normal human keratinocytes (NHKs) were purchased (Kurabo Industries, Osaka, Japan). NHK cells were derived from neonatal foreskin. HaCaT cell line, which is spontaneously immortalized normal human adult skin keratinocyte, [26] was a kind gift from Dr. NE Fusenig (University of Ulm, Ulm, Germany). The human SCC cell line DJM-1, which was derived from malignant trichilemmal cyst of a Japanese woman [27], was a kind gift from Dr. Y. Kitajima (Department of Dermatology, Kizawa Memorial Hospital, Gifu, Japan). The human SCC cell line KU-8, established from a lymph node metastasis of penile skin SCC, [28], was a kind gift from T. Tsukamoto (Department of Urology, School of Medicine, Keio University, Tokyo, Japan). The human SCC cell line A431 (derived from epidermoid carcinoma) and HeLa cells (derived from cervical cancer) were purchased (DS Pharma Biomedical, Osaka, Japan). Human fibroblasts were purchased (Cell Applications Inc., San Diego, CA) and used between passage 3 and 5. Apart from NHK and CHO-K1 cells, all other cells were cultured in DMEM medium supplemented with 10% FCS.

### Indirect immunofluorescence of normal human skin

Normal human skin samples from various anatomical locations were obtained from 5 Asian (Japanese) individuals, whereas one Caucasian sample was purchased (Biochain, Hayward, CA). One Asian sample was processed as frozen while the rest were formalin-fixed and paraffin-embedded (FFPE). FFPE sections were deparaffinized and dehydrated. Antigen retrieval for FFPE sections was performed by autoclaving in 10mM citrate buffer, pH 6.0. Both frozen and FFPE skin sections were blocked with 1% BSA/PBS for 30 min and incubated with 4 μg/ml of anti-CD44 monoclonal antibodies (mAb) in 1% BSA/PBS at room temperature for 1 hour. Antibodies were against CD44s, clone 156-3C11 (Lab Vision, Fremont, CA), CD44v6, clone 2F10 (R&D Systems, Minneapolis, MN) and CD44v9, clone RV3 [29]. Normal mouse IgG (Invitrogen, Carlsbad, CA) was used as negative control. Alexa Fluor 488-conjugated anti-mouse or anti-rat IgG (Invitrogen) diluted 1: 1000 were used as secondary antibodies. Nuclei were counter-stained with 0.4 μg/ml 4’,6-diamidino-2-phenylindole (DAPI) (Nacalai Tesque, Kyoto, Japan).

### RNA isolation from human epidermis and skin and cDNA synthesis

To make epidermal cDNA, unwanted human adult foreskin, obtained after circumcision, was separated into epidermis and dermis by treatment with sodium thiocyanate [30,31]. Epidermis was homogenized with Biomasher II and total RNA was isolated with RNeasy Kit (Qiagen, Hilden, Germany). Whole human skin samples from 2 additional Asian donors were homogenized and RNA was isolated as described above. cDNA was synthesized with SuperScript III First-Strand Synthesis System (Invitrogen). Whole human skin cDNA from a non-Japanese Asian was purchased (Biochain, Hayward, CA).

### PCR and cloning of *CD44* transcripts

We designed PCR primers for *CD44* transcripts with 14–15 bp 5’ extensions that are complementary to the ends of *Hind*III- and *Xho*I-digested pcDNA-myc-his vector. The sequences of PCR primers were F; TGGCTAGTTAAGCTTGCCACCATGGACAAGTTTTG and R; GCCCTCTAGACTCGAGCACCCCAATCTTCATGTCC. Underlines indicate sequences specific to *CD44* and non-underlined parts indicate extension sequences homologous to vector ends. PCR was performed with PrimeSTAR Max DNA polymerase (Takara Bio, Shiga, Japan) following the manufacturer’s guidelines. PCR products were separated by both 1% agarose and 5–20% gradient acrylamide (ATTO, Nagoya, Japan) gels. Several portions of both gels were cut, extracted separately and recovered DNA fragments were cloned directly into the above vector by using In-Fusion Cloning HD Kit (Clontech Laboratories, Mountain View, CA). Inserts were analyzed by PCR and many clones were sequenced completely.

### Transfection of CHO-K1 cells and immunofluorescence analysis

CHO-K1 cells grown on cover glass were transfected with each *pcDNA/CD44* construct with ScreenFect A transfection reagent (Wako Pure Chemical Industries, Osaka, Japan) following the manufacturer’s instructions. After culturing for 2 days, the cells were fixed with cold methanol for 10 min at -30°C, blocked with 1% BSA in PBS for 30 min and incubated with Alexa Fluor 488-conjugated anti-His tag antibody (MBL, Nagoya, Japan) for 1 hour at room temperature. After washing 3 times with PBS, cells were observed with a BX51 fluorescence microscope (Olympus, Tokyo, Japan).

### Cell culture and treatments

NHKs were cultured in EpiLife medium supplemented with HKGS (Invitrogen) under low calcium conditions. For differentiation, cells were switched to EpiLife medium without HKGS under 2 mM CaCl_2_ with or without 5% FCS. Samples were taken at indicated time points for RNA extraction and cDNA synthesis. For treatment with chemicals, cells were then switched to EpiLife medium with or without 2 mM CaCl_2_ and supplemented with various agents that are known affect skin barrier function. After culturing for further 3 days, samples were taken for RNA extraction, cDNA synthesis and PCR analysis of *CD44* transcripts. The concentrations of the agents are 20 ng/ml epidermal growth factor (EGF) and 20 ng/ml TGF-β1 (Pepro Tech, Rocky Hill, NJ), 20 ng/ml basic FGF (DS Pharma Biomedical), 100 μM hydrogen peroxide, 50 ng/ml retinoic acid (RA) and 30 μM vitamin D3 (Wako), 20 ng/ml IL-17 (HumanZyme, Chicago, IL), 0.5 μg/ml Plasmin (Acris Antibodies, Herford, Germany), 5% FCS, and 25 ng/ml phorbol 12-myristate 13-acetate (PMA) (LC Laboratories, Woburn, MA). Medium or DMSO (0.1% final concentration) were used as mock treatments.

DJM-1 cells were cultured in complete EpiLife medium for more than 8 days before used for experiments. DJM-1 and NHK cells were seeded into 6-well plates and cultured in EpiLife medium until 70% confluency before various treatments were performed. Briefly, medium was switched to EpiLife without HKGS, and supplemented with calcium and with or without 5% FCS. Cells without FCS were cultured for 2 and 4 days before harvesting. Some cells with FCS were cultured for 2 and 4 days before harvesting. For some cells with FCS, after 2 days, FCS was removed and cells were further cultured for 2, 4 and 6 days before harvesting for RNA extraction using GenElute Mammalian Total RNA Kit (Sigma-Aldrich, St. Louis, MO) and cDNA synthesis using PrimeScript II cDNA Kit (Takara). PCR was performed as described above for *CD44* transcript analysis.

## Results

### Detection of CD44 in normal human skin by immunofluorescence

**Fig 1:**
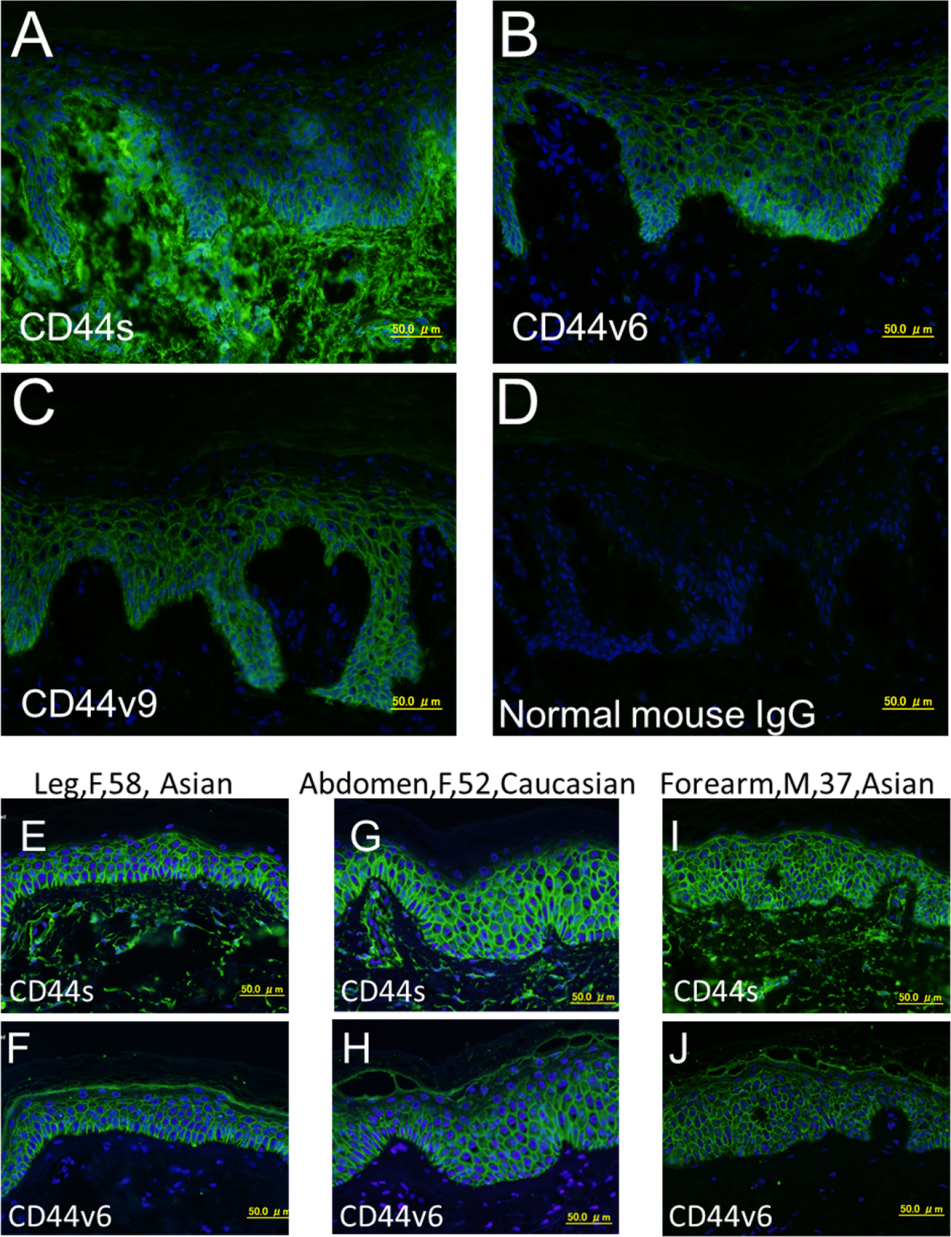
Immunofluorescence analysis of CD44 expression in frozen and FFPE normal human skin. (A) CD44s-specific mAb stained both epidermis and dermis of frozen human skin. (B, C) Only epidermis of frozen human skin was stained by CD44v6-specific mAb (B) and CD44v9-specific mAb (C). (D) Normal mouse IgG (negative control) showed no signal in either epidermis or dermis frozen human skin. (E-J) Analysis of CD44s and CD44v6 expression in FFPE samples from different anatomical locations, gender, age and ethnicity. Nuclei were counterstained with DAPI. Scale bars, 50 μm.

To clarify the expression of CD44 in human skin, we first examined the expression patterns of CD44s, and v6- and v9-containing CD44 isoforms in various normal human skin sections by immunofluorescence analysis. CD44s was expressed prominently in both epidermis and dermis of frozen skin section (Fig 1A). In contrast, v6- and v9-containing isoforms were expressed exclusively on cell surfaces in epidermis (Fig 1B and 1C). In these analyses, no staining was observed when normal mouse IgG was used instead of primary antibodies (Fig 1D). We also found that the expression pattern of CD44 is similar in subjects with different age, location of skin, gender and ethnic background (Fig 1E–1J and Table 1). These results suggested expression pattern of CD44 variants is universal regardless of the anatomical location of skin, gender, age and ethnicity. The results also revealed that human epidermis has more CD44 diversity than dermis. Therefore, we focused on human epidermis in the following studies.

**Table 1:** Summary of analysis of CD44s, CD44v6 and CD44v9 expression in frozen and FFPE normal human skin.

#	Location	Gender	Age	Ethnicity	Type	CD44s	CD44v6	CD44v9
1	NA^1^	NA^1^	NA^1^	Asian	Frozen	Whole skin	Epidermis	Epidermis
2	Abdomen	Female	42	Asian	FFPE	Whole skin	Epidermis	Epidermis
3	Leg	Female	58	Asian	FFPE	Whole skin	Epidermis	Epidermis
4	Forearm	Male	37	Asian	FFPE	Whole skin	Epidermis	Epidermis
5	Thigh	Female	66	Asian	FFPE	Whole skin	Epidermis	Epidermis
6	Abdomen	Female	52	Caucasian	FFPE	Whole skin	Epidermis	Epidermis

^1^Not Available

### Detection of *CD44* transcripts in human epidermis

**Fig 2:**
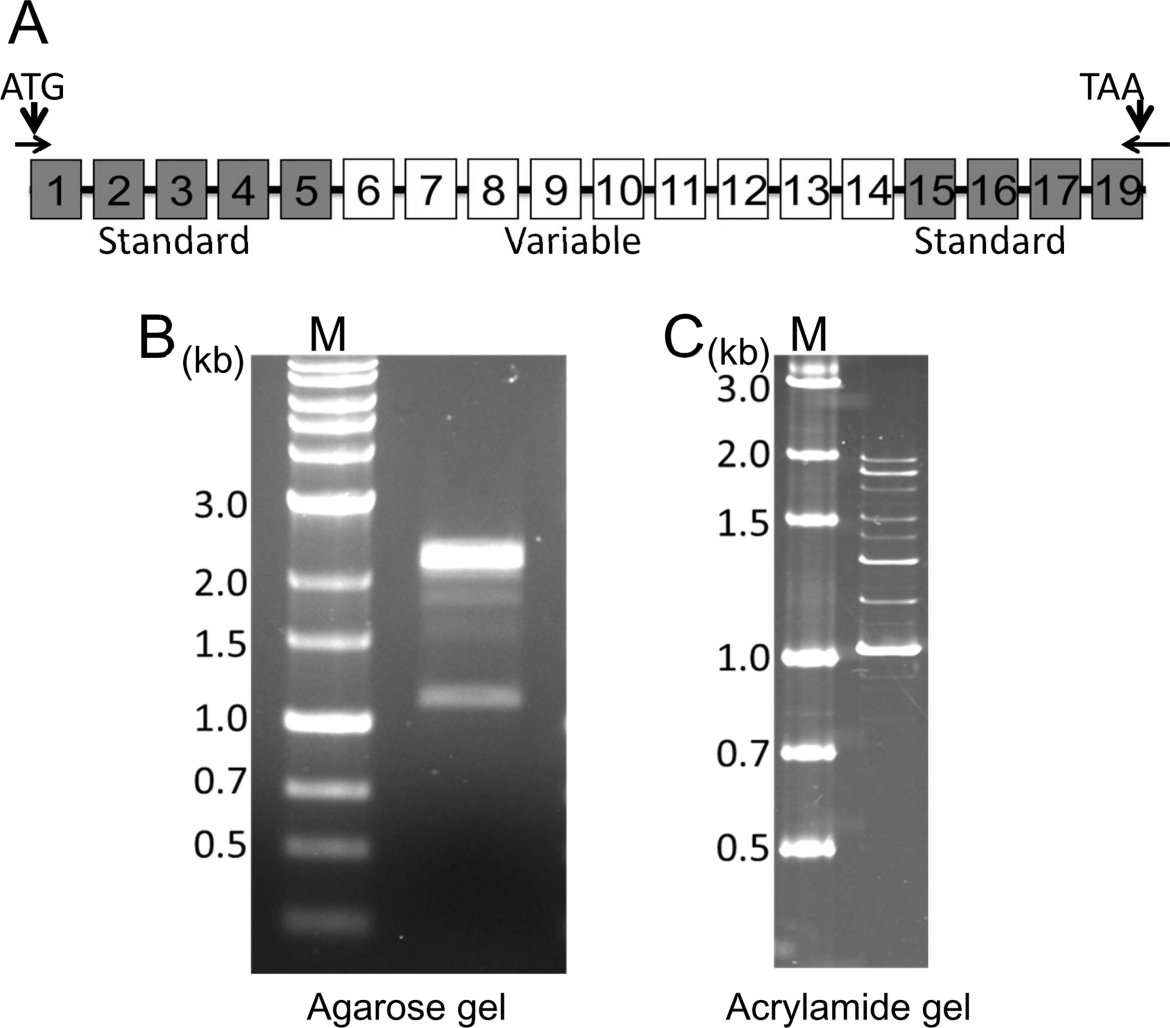
PCR analysis of *CD44* transcripts in human epidermis. (A) PCR strategy. Primers were designed to amplify all variants of full-length *CD44* transcripts in human epidermis. Filled boxes represent standard exons and empty boxes represent alternatively spliced exons. The non-coding exon 18 is not shown. (B, C) Analysis of PCR products of *CD44* transcripts on 1% agarose gel (B) and 5–20% acrylamide gel (C).

We next isolated RNA from human epidermis and produced cDNA pool by reverse transcription. cDNA products were obtained by PCR with primers designed to amplify “full-length” *CD44* transcripts (Fig 2A). By agarose gel electrophoresis, we observed multiple bands, suggesting that a number of *CD44* transcripts with different exon combinations were expressed in human epidermis (Fig 2B). To clearly demonstrate the number of *CD44* transcripts in human epidermis, we separated the PCR product by acrylamide gel electrophoresis, which can separate DNA fragments with a higher resolution than agarose gel. This method showed that at least 10 *CD44* transcripts were variably expressed in human epidermis (Fig 2C).

### Cloning of *CD44* transcripts in human epidermis

**Fig 3:**
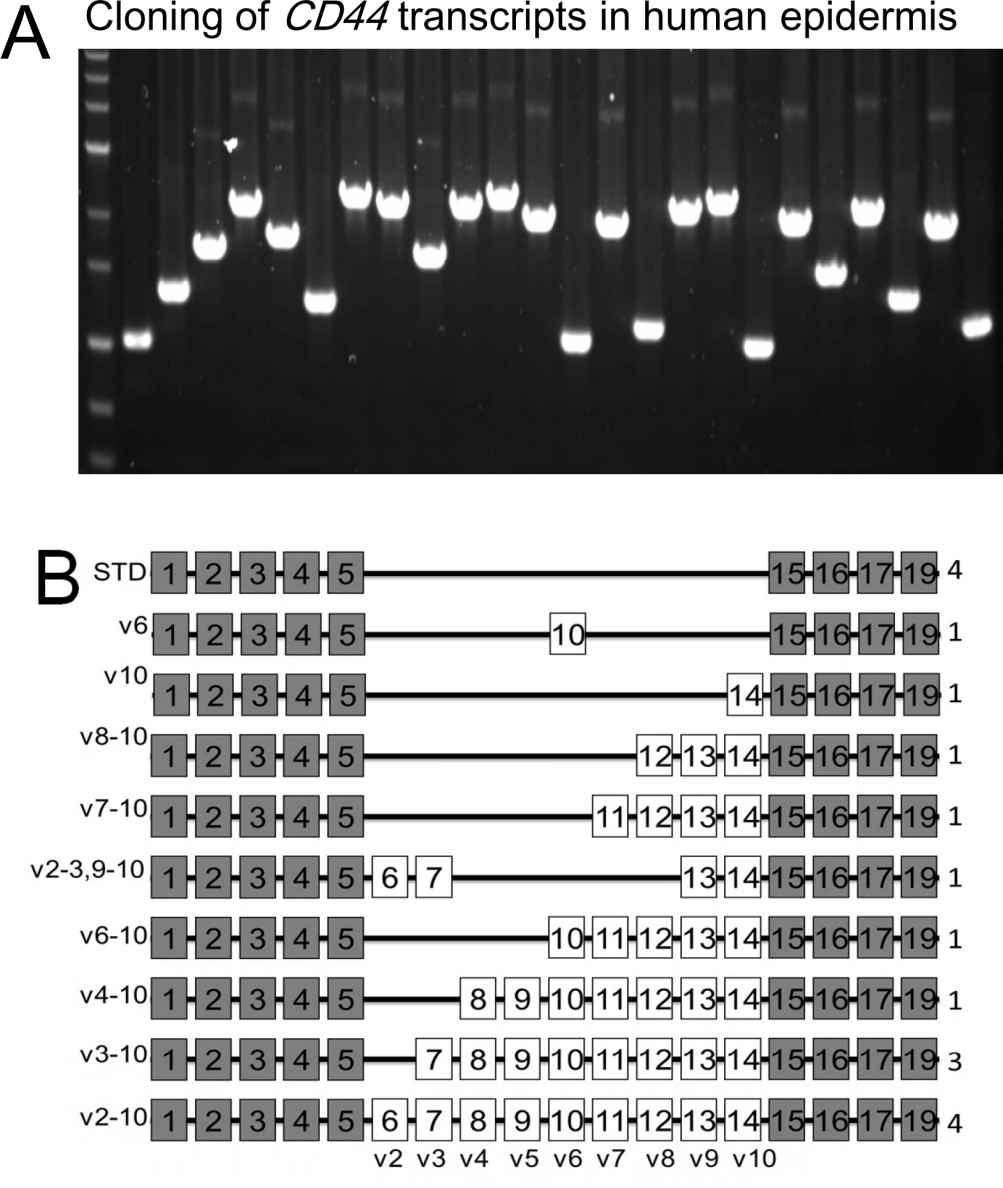
Cloning of *CD44* transcripts in human epidermis. (A) Successful cloning of different *CD44* transcripts from human epidermis by PCR. (B) Summary of *CD44* transcripts in human epidermis; Epidermis contains at least 10 major classes of *CD44* transcripts. The numbers on the right indicate the number of variants in each class.

To study extensively the nature of *CD44* transcripts, which are expressed in human epidermis, we cloned *CD44* cDNA fragments recovered from both agarose and polyacrylamide gels. To avoid the digestion of unknown sequences by restriction enzymes, we used in-fusion cloning technology (Clontech Laboratories), which is based on complementary pairing of DNA ends, rather than ligation, without restriction digestion of PCR product. DNA fragments were cloned directionally into pre-linearized pcDNA-myc-his vector (Invitrogen). PCR analysis of the resultant cDNA clones revealed successful cloning of different *CD44* transcripts from human epidermis (Fig 3A).

### Identification of *CD44* transcripts in human epidermis

**Fig 4:**
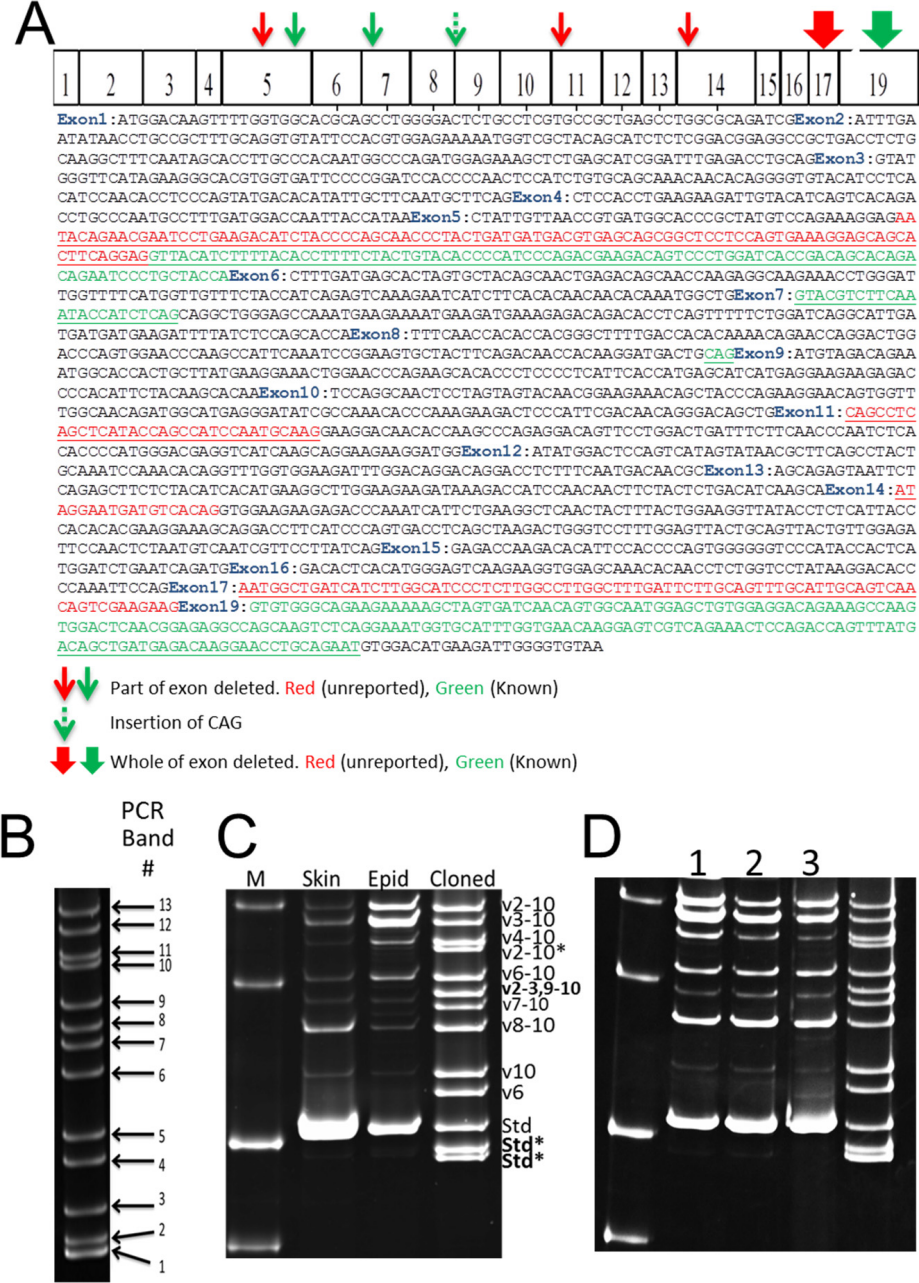
Analysis of *CD44* transcripts in human epidermis. (A) Presentation of *CD44* cDNA structure and sequences with features reported and those identified in this study. (B) Identification of PCR bands of cloned *CD44* transcripts in human epidermis. PCR bands numbers corresponding to *CD44* transcripts are indicated. (C) Analysis of PCR products of skin and epidermal cDNA in acrylamide gel and comparison of sizes of native *CD44* transcripts with cloned *CD44* transcripts. Transcripts with * are missing some sequences. Previously unreported forms are shown in bold characters. (D) Analysis of *CD44* transcripts in human skin obtained from subjects with different anatomical location, gender, age and ethnicity. Samples were taken from: 1, Japanese, female, face, 97; 2, Japanese, male, finger, 71; 3, non-Japanese Asian, male, 24, location unknown.

To reveal precise structures of all *CD44* transcripts in human epidermis, all the resultant cDNA clones were entirely sequenced. We found that 10 major classes of *CD44* transcripts were expressed in human epidermis (Fig 3B). Detailed sequence analysis showed that some transcripts showed variations by loss of whole exons or their parts (Table 2 and Fig 4A). Importantly, we found 3 previously unreported transcripts. We found 2 unreported transcripts, which were smaller than *CD44s*. These transcripts lost either a part of exon 5 or whole exon 17 (Table 2). Additionally, we found one reported *CD44s* transcript lacking part of exon 5. The absence of exon 17 creates a pre-mature termination codon within exon 19, resulting in a CD44s protein without transmembrane and cytoplasmic domains. We also found one non-*CD44s* transcript, which missed exon 19. This transcript was predicted to encode a CD44 protein without cytoplasmic domain. An unreported transcript, *CD44v2-3*, *9–10*, was the only transcript with non-continuous alternatively spliced exons in human epidermis. *CD44v6* and *CD44v10* were the only transcripts with single alternatively spliced exons inserted within the *CD44s* transcript. Furthermore, we found insertion of CAG trinucleotide between exon 8 and 9 in some transcripts (Table 2). Thus, our study showed that there were 18 *CD44* transcripts in human epidermis (Table 2 and Fig 4B).

**Table 2:** Characteristics of the 18 *CD44* transcripts in human epidermis.

Transcript number	Variant exon number									Exon 17	Exon 19	CAG^1^	Bp	Protein Class	PCR Band number
	6	7	8	9	10	11	12	13	14						
1	-	-	-	-	-	-	-	-	-	+	+	N/A	990	**Std**^2^	1
2	-	-	-	-	-	-	-	-	-	+	+	N/A	993	Std^3^	1
3	-	-	-	-	-	-	-	-	-	-	+	N/A	1007	**Std**^4^	2
4	-	-	-	-	-	-	-	-	-	+	+	N/A	1086	Std	3
5	-	-	-	-	+	-	-	-	-	+	+	N/A	1215	v6	4
6	-	-	-	-	-	-	-	-	+	+	+	N/A	1290	v10	5
7	-	-	-	-	-	-	+	+	+	+	+	N/A	1482	v8-10	6
8	-	-	-	-	-	+^5^	+	+	+	+	+	N/A	1581	v7-10	7
9	+	+	-	-	-	-	-	+	+	+	+	N/A	1635	**v2-3,9–10**	8
10	-	-	-	-	+	+	+	+	+	+	+	N/A	1743	v6-10	9
11	-	+	+	+	+	+	+	+	+	+	-	+	1921	v2-10	10
12	-	-	+	+	+	+	+	+	+	+	+	+	1977	v4-10	11
13	-	+	+	+	+	+	+	+	+^6^	+	+	+	2085	v3-10	12
14	-	+	+	+	+	+	+	+	+	+	+	-	2100	v3-10	12
15	-	+	+	+	+	+	+	+	+	+	+	+	2103	v3-10	12
16	+	+^7^	+	+	+	+	+	+	+	+	+	+	2208	v2-10	13
17	+	+	+	+	+	+	+	+	+^6^	+	+	+	2214	v2-10	13
18	+	+	+	+	+	+	+	+	+	+	+	+	2232	v2-10	13

^1^Extra CAG trinucleotide between exon 8 and 9, N/A, Not applicable

^2^Deletion of 96 bases within exon 5, **previously unreported,** Std, Standard

^3^Deletion of last 93 bases of exon 5

^4^Deletion of exon 17, **previously unreported**

^5^Deletion of first 33 bases

^6^Deletion of first 18 bases

^7^Deletion of first 24 bases

Previously unreported sequences are shown in bold

### Expression of *CD44* transcripts in human whole skin compared to epidermis

Acrylamide gel analysis of cDNA products of both human whole skin and separated epidermis revealed more than 10 *CD44* transcripts for both tissues (Fig 4C). The number of transcripts was similar in both tissues, although expression levels varied significantly. We could isolate and designate all major *CD44* transcripts in whole human skin and epidermis by comparing the sizes of their PCR products to those obtained from the cloned *CD44* transcripts (Fig 4B and 4C and Table 2). In whole skin, *CD44s* appeared to be the major transcript, whereas *CD44s*, *CD44v3-10* and *CD44v2-10* were the major transcripts in epidermis (Fig 4C). Moreover, as also observed by immunofluorescence, *CD44* transcript expression in whole human skin is not significantly influenced by anatomical location, age, gender and ethnicity (Fig 4D).

### Protein expression induced by cloned *CD44* transcripts

**Fig 5:**
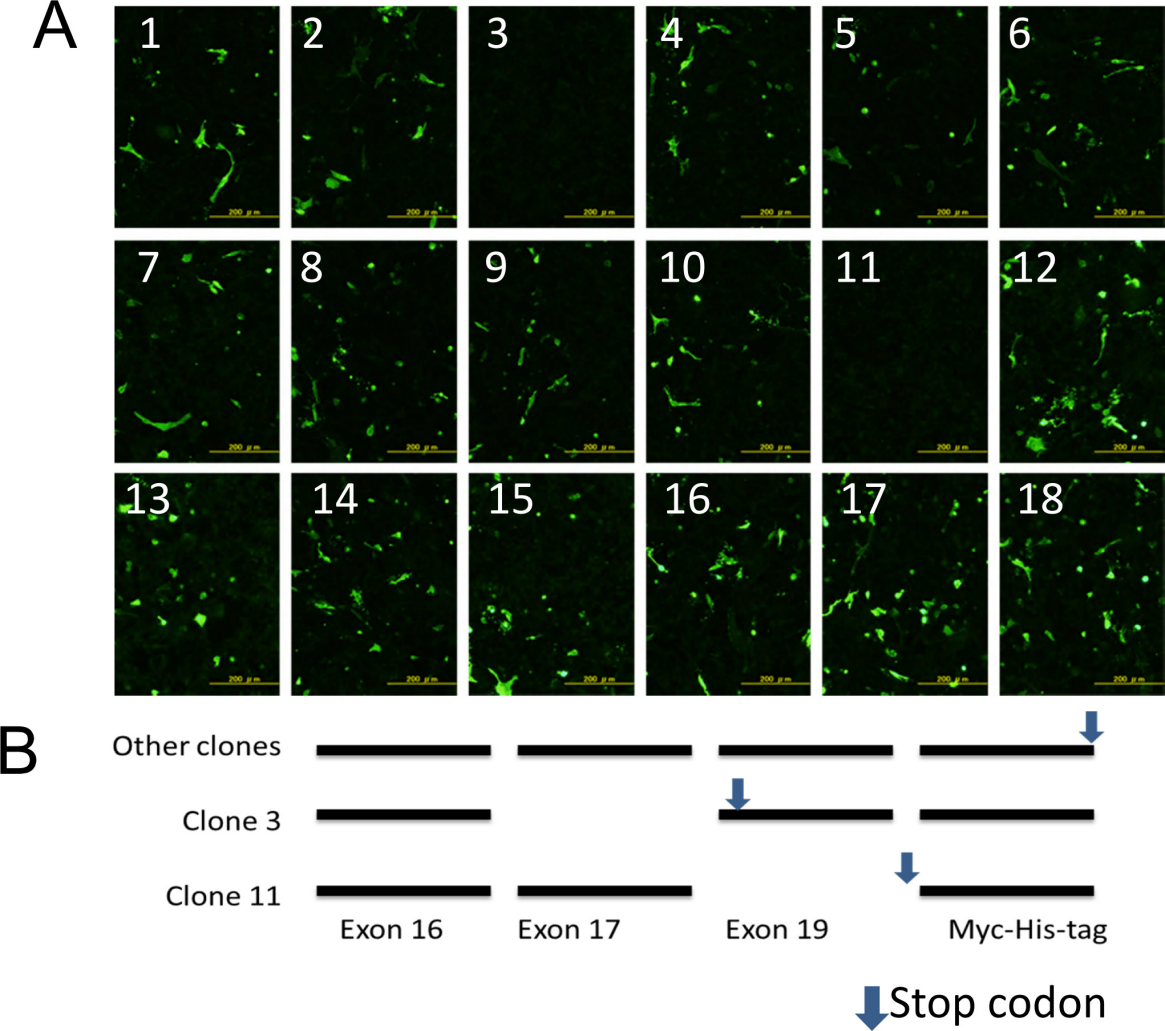
Protein expression induced by cloned *CD44* transcripts. (A) Immunofluorescence analysis of stable protein expression by cloned *CD44* transcripts in transfected CHO-K1 cells. The expressed proteins were detected with an antibody to His-tag. Scale bars, 200 μm. (B) Clones 3 and 11 had stop codons before His-tag.

We next examined whether the *CD44* transcripts stably expressed proteins. We transfected each clone into CHO-K1 cells and examined protein expression by immunofluorescence. Most clones stably expressed recombinant proteins that could be detected with anti-His-tag antibody (Fig 5A). Only clones 3 and 11 expressed no protein. Our further analysis showed that clones 3 and 11 lacked exon 17 and 19, respectively (Fig 5B). These missing sequences would result in pre-mature termination codons, depriving the synthesized proteins of the polyhistidine His-tag epitope used for detection in the immunofluorescence study. At least 88.9% of the 18 epidermal *CD44* transcripts stably produced proteins in transfected CHO-K1 cells.

### Regulation of *CD44* transcript expression by various agents

**Fig 6:**
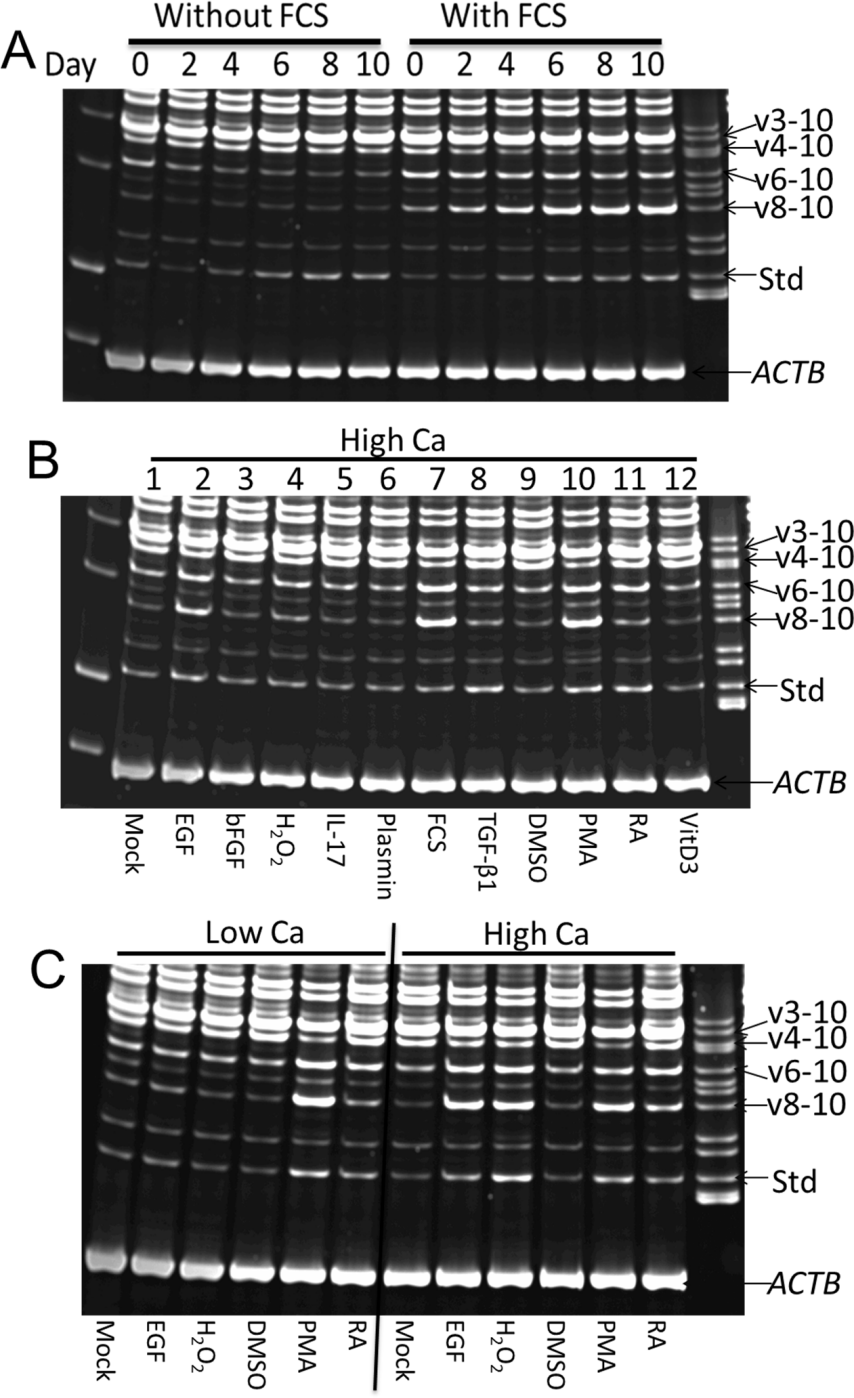
Analysis of regulation of variant *CD44* transcript expression by various agents in NHK cultures. (A) After low to high calcium switch with or without FCS, cells were cultured for the indicated number of days. With exception of day 0, all cells were cultured in high calcium medium. (B) NHKs were cultured in high calcium medium with or without various agents. H_2_O_2_ = hydrogen peroxide. (C) NHKs were cultured in low or high calcium medium with or without selected agents. All experiments were performed 3 times and representative results are shown.

We next examined the regulation of expression of *CD44* transcripts in cultured NHKs. First, we investigated calcium-induced keratinocyte differentiation in the presence and absence of FCS. Most transcripts were differentially regulated during calcium-induced keratinocyte differentiation both in the presence and absence of FCS (Fig 6A). Transcript *v3-10* remained the major form of *CD44* expressed by NHKs in all tested conditions. However, cultivation of keratinocytes in the presence of FCS and calcium, but not with calcium alone, resulted in progressive, time-dependent up-regulation of the *CDv8-10* variant (Fig 6A). Furthermore, expression of *CD44v6-10* appeared to decline during calcium-induced keratinocyte differentiation in the absence of FCS (Fig 6A). On the other hand, the expression of *CD44v6-10* was increased in a time-independent manner during calcium-induced keratinocyte differentiation in the presence of FCS (Fig 6A). *CD44s* showed progressive time-dependent up-regulation during calcium-induced keratinocyte differentiation both in the presence and absence of FCS. Next, we examined the effects of various skin biology-related agents that are known to affect skin barrier function on regulation of expression of *CD44* variants in NHKs. Several agents, particularly EGF, hydrogen peroxide, PMA, RA and FCS differentially regulated *CD44* variant expression and these effects were partly dependent on calcium (Fig 6B and 6C). In these analyses, at least 5 *CD44* transcripts were clearly and differentially regulated under different cell culture conditions. *CD44v8-10* isoform was up-regulated again in the presence of FCS and calcium and by exposure to EGF plus Ca^2+^ or to PMA (Ca^2+^-independent), whereas *CD44v3-10* remained invariably high, although it was slightly down-regulated by PMA (Fig 6C). *CD44v6-10* was also up-regulated by EGF (Ca^2+^-dependent), FCS, H_2_O_2_ (Ca^2+^-dependent), PMA (Ca^2+^-independent) and RA (Ca^2+^-independent) (Fig 6B and 6C). PMA and RA also up-regulated *CD44s* in a Ca^2+^-independent manner whereas EGF and H_2_O_2_ upregulated *CD44s* in a Ca^2+^-dependent manner.

### Diverse expression patterns of *CD44* transcripts among different human normal and malignant keratinocytes

**Fig 7:**
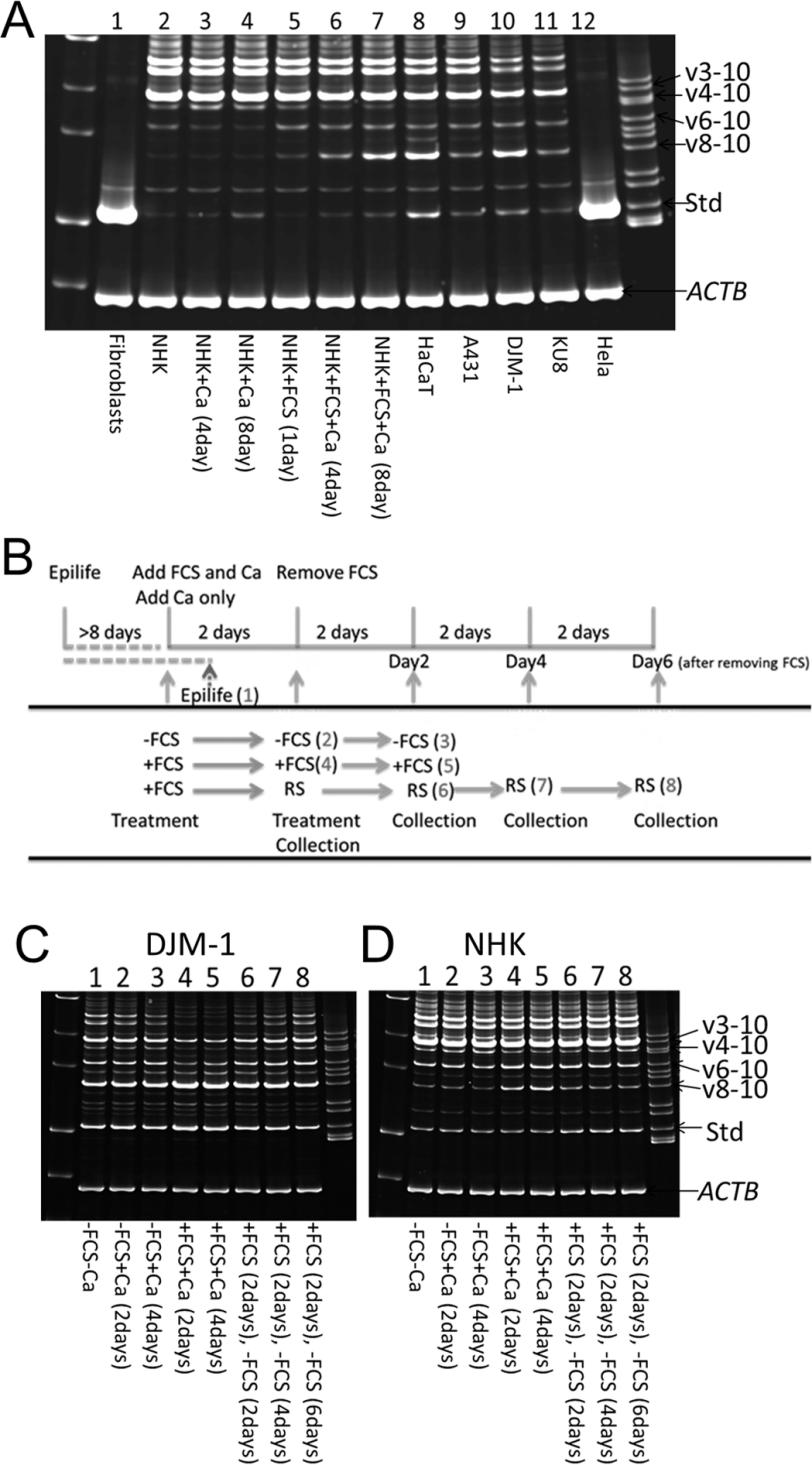
Analysis of *CD44* transcripts in several cell types. (A) *CD44* transcripts in different cells. (B) Experimental design for analysis of regulation of *CD44* expression in normal and malignant keratinocytes by FCS. Cells were treated and harvested at the indicated time points. Numbers in parenthesis correspond to the lane numbers in C and D below. (C, D) Analysis of *CD44* transcripts in DJM-1 (C) and NHK (D) cells cultured with or without FCS containing or not CaCl_2_. All experiments were performed 3 times and representative results are shown.

We next compared the expression of *CD44* transcripts between NHKs and other cultured cells, and found that expression pattern of *CD44* in NHKs differed from those in other cells (Fig 7A). Human dermal fibroblasts and HeLa human cervical carcinoma cell line predominantly expressed *CD44s*. *CD44* transcription in other squamous cell carcinoma (SCC) cell lines, including KU8, DJM-1 and A431, was similar to that observed in the spontaneously immortalized normal human keratinocyte line HaCaT and in the NHKs cultured with FCS, although *CD44s* expression remained unchained. In keratinocyte cell lines, like in NHKs, the predominant form of expressed *CD44* was *v3-10*. Additionally, DJM-1 and HaCaT lines showed an increased expression of *CD44 v8-10* (Fig 7A).

### FCS differently regulates *CD44* transcription between NHKs and SCC cells

The results from regulation of *CD44* transcription by various agents clearly showed that a number of *CD44* transcripts in NHKs were regulated by FCS. Moreover, NHKs treated with FCS, in the presence of calcium, showed similar expression of *CD44* transcripts as several tumor cells. To identify *CD44* transcripts that segregate with mitogenic or tumor-related activities, we first adapted DJM-1 cells, a SCC cell line, to serum-free condition, as we also used for NHKs (Fig 7B). We then examined expression of *CD44* transcripts, after DJM-1 cells were treated with FCS for 2 days. Expression of *CD44* was examined 2, 4 and 6 days after FCS was removed (Fig 7B). This analysis showed striking differences in response to FCS supplementation and FCS withdrawal between NHKs and DJM-1 cells (Fig 7C and 7D). Amount of *CD44v3-10* was high and was not significantly influenced by FCS treatment in NHKs. In contrast, the amount of *CD44v3-10* was low and visibly decreased by FCS treatment in DJM-1 cells. Amount of *CD44v8-10* was high in DJM-1 cells when compared to NHKs and treatment with FCS, in the presence of calcium, up-regulated *CD44v8-10* in both cell types (Fig 7C and 7D). FCS removal down-regulated *CD44v8-10* more efficiently in NHKs than in DJM-1 cells. Furthermore, a relatively higher expression of *CD44s* in DJM-1 cells declined slightly after FCS withdrawal. On the contrary, *CD44s* amount in NHKs appeared to increase after FCS removal (Fig 7C and 7D).

## Discussion

This is the first study for complete isolation and designation of all *CD44* transcripts in epidermis and skin. Our cloning strategy showed that various *CD44* forms with alternatively spliced exons are expressed in human epidermis. Dermal fibroblasts predominantly expressed *CD44s* transcript, as also visualized with immunofluorescence at protein level in the dermis. More diversity was noted in epidermis, where we have identified 18 unique *CD44* transcripts. Although more than 800 different *CD44* forms are theoretically possible [15], the diversity in epidermis compared to dermis was remarkable.

When cloning mixtures of DNA fragments into vectors, efficiency is higher for highly expressed transcripts and smaller transcripts. By cutting different bands containing each PCR product from acrylamide and agarose gels and cloning them separately, we were able obtain all possible *CD44* cDNA in human epidermis, including those with low abundance. Use of acrylamide gel resolution of PCR products permitted more efficient separation, which allowed us to successfully identify all *CD44* transcripts in human epidermis. Previous attempts using PCR methods were based on PCR product size calculation, but this approach could not fully assign identities to all bands [15]. Thus, in calculation-based methods, absence or presence of particular nucleotides is inaccurate, and the presence of multiple bands makes direct DNA sequencing impossible.

In this study, we identified a previously unreported *CD44v2-3*,*9–10* transcript. The variable v3 domain of CD44 protein harbors attachment site for heparan sulfate GAG [32], whereas dermatan/chondroitin sulfate GAG decorate invariable N-terminal domain [3]. Attachment of GAGs was demonstrated to enhance the presentation of growth factors, including heparin-binding growth factor [33], and this mechanism is important during certain physiological and disease processes including control of dermatoporosis (chronologic skin aging) [34–36], wound healing [37], keratinocyte migration [37] and tumor development and metastasis [38,39]. Despite its relatively low level of transcription, *CD44v2-3*,*9–10* was up-regulated by PMA, in the presence of high calcium, by FCS, EGF and H_2_O_2_. In DJM-1 cell line, which showed higher expression of *CD44v2-3*,*9–10* than NHKs, this *CD44* variant showed the same up- and down- regulation upon FCS switch, as *CD44v8-10* in normal keratinocytes. *CD44v2-3*,*9–10*, the smallest *CD44* transcripts containing v3, may possess strong growth factor presentation ability due to its smaller size and may be important in the skin.

In this study, we have selected epidermis, specifically, for several reasons: (i) CD44 is the cell surface receptor for HA and HA is the major component of the epidermal intercellular matrix involved in the nutrition of this non-vascularized tissue, in regulation of the gradients of ions and growth factors, and in promotion of keratinocyte responses to wound healing stimuli [40]. (ii) CD44 isoforms are suggested to be involved in epidermal tumorigenesis and stem cell regulation [41–43]. (iii) We are currently studying the exact nature and expression of a novel keratinocyte antigen, desmosealin, which we believe being related to the CD44 family of proteoglycans [44]. In this context, it appeared important to define all existing transcripts of the *CD44* gene in human keratinocytes, which should permit further studies on potential roles of these various proteins in keratinocyte biology, formation of the epidermal permeability barrier, and epidermal carcinogenesis. (iv) Also as we stated at the beginning of the results section, epidermis appears to show much diversity of *CD44* transcripts and should serve as good source of information for CD44 expression.

Previous studies on CD44 and epidermal differentiation defined CD44v3-10, also called epican, as the most abundant and largest CD44 isoform in human epidermis [45–47]. However, our study demonstrate an abundant *CD44v2-10* transcript, which is larger than *CD44v3-10*. This was made possible by our use of PAGE for DNA separation. CD44v3-10 mediates keratinocyte cell-cell adhesion via hyarunonan-dependent mechanism [46]. This desmosome-independent way of keratinocyte interaction is made possible by the presence of a specific hyaluronan-binding sequence on the standard portion of CD44 common to all isoforms [48,49]. Expression of four CD44 isoforms, namely CD44s, CD44v8-10, CD44v3-10, and an ill-defined 180kDa CD44 isoform, was demonstrated to depend on the keratinocyte growth potential and degree of their terminal differentiation. Only the latter two CD44 proteins persisted on differentiated cells [47]. The CD44v8-10 isoform, previously denoted as CD44E, was also described in colon cancer and leukemia cells [48,50]. Although it is deprived of the v3 segment, where heparan sulfate GAG side chain is situated, expression of this isoform segregates with keratinocyte growth and not with differentiation [47]. Also in our study, we observed a higher constitutive expression of CD44v8-10 in immortalized HaCaT and SCC-derived DJM-1 cell lines and its up-regulation by EGF, FCS and PMA phorbol ester. Interestingly, some CD44 variants, particularly CD44v8-10 were shown to regulate redox status in cancer cells, and promoted tumor growth [29,51,52]. Therefore, specific expression of normal amount of CD44v8-10 may be required for proper maintenance of the redox status in human epidermis, which is exposed constantly to mechanical stress and ultraviolet irradiation.

In this study, we investigated the effects of several agents on regulation of *CD44* transcript expression in cultured human keratinocytes. These agents were chosen because they are known to affect various pathways and/or processes in mouse and human skin *in vivo* and/or *in vitro*. Both EGF and basic FGF are involved in signaling through the Ras/Raf/MAPK and PI3K/Akt pathways via different mechanisms to control gene expression, cellular proliferation, cell motility, differentiation and inhibition of apoptosis, which contributes to tumor development [53–56]. Basic FGF has also been shown to be involved in wound healing [57,58] and DNA repair [59]. PMA is regarded as a potent tumor promoter and it is often used to activate the protein kinase C (PKC) pathway, which is involved in keratinocyte proliferation and differentiation [60,61]. RA signaling via retinoic acid receptor, has been shown to play a role in skin cell proliferation, differentiation, apoptosis, and epidermal barrier function and it is often used for treatment of skin aging [62–65]. Calcium has been shown to up-regulate differentiation- and ribosome protein-related genes and down-regulate genes involved in metabolism, DNA repair, transcription, and translation in primary human keratinocytes [66]. FCS is known to contain many mitogenic and growth factors and it is used to stimulate growth of many cell types [67]. Plasmin has been shown to be involved in wound healing by promoting migration of epidermal keratinocytes coupled with enhanced phagocytic-killing and inhibition of proliferation, which may facilitate re-epithelialization following skin injury [68] and has been shown to play a role in the amplification of psoriasiform skin inflammation in mice [69]. H_2_O_2_ has been demonstrated to be involved in wound healing [70], UV-induced DNA damage in keratinocytes [71], and skin blanching [72], IL-17 is a critical component of the Il-17/Il-23 pathway, which is involved skin inflammation and pathogenesis of psoriasis [73–76]. TGFβ1 has been shown to be involved in control of wound healing and inhibition of keratinocyte proliferation [77]. In addition, TGFβ1 has been shown to regulate NFκB dependent gene expression in mouse keratinocytes, which is important in regulation of epidermal homeostasis, inflammatory responses and carcinogenesis [78]. VitD3 pathway is active in skin and it has been shown to be involved in differentiation of keratinocytes via the PKC pathway [79–81]. VitD3 has also been shown to affect expression of genes in skin and topical application of VitD3 and its analogs can improve hyperproliferative skin diseases like psoriasis [82]. Previously unreported, our study indicated that at least 5 *CD44* transcripts were differentially regulated by various agents, particularly calcium, FCS, EGF, hydrogen peroxide and PMA, which target cell differentiation, proliferation (FCS and EGF), oxidative stress and neoplastic transformation, respectively. Further to the effects of these chemical agents on *CD44* expression, we demonstrated that some transcripts were expressed differently between normal and tumoral keratinocytes. These results suggested that particular *CD44* variants play an important role in the homeostasis of human epidermis, possibly via some specific biological pathways. Specifically, proper balance among *CD44*s, *CD44v8-10* and *CD44v3-10* maintains epidermal homeostasis, and its dysregulation causes epithelial tumor development. One limitation of our study is that, the normal and tumor cells were not derived from the same source. Nevertheless, we demonstrated that normal skin from different anatomical location, gender, age and ethnicity show similar expression pattern of both protein and transcripts. Thus, keratinocytes from any individual and anatomical location may represent a good source of information about CD44 expression. Future studies using normal and tumor keratinocytes from the same subject may provide even more reliable information about the regulation of CD44 expression by extracellular agents.

Future studies are needed to fully understand functions of the 18 CD44 variants in cell survival, cell proliferation or resistance to apoptosis in human epidermis. Their functions should be influenced not only by the peptide sequences but also by the nature, position and quantity of glycans decorating their external portions [44,83–85].

Because we revealed all *CD44* transcripts in human epidermis, it is now possible to design specific assays to examine the presence and fates of these transcripts in normal and disease states. Our study will serve as a useful guide in this respect. Previous reports suggested that CD44v6 was highly expressed in various human tumors and was a target for therapy by a monoclonal antibody [19]. However, severe skin toxicity hampered development of the therapy [86,87]. Our comprehensive analysis demonstrated that more than half of the *CD44* transcripts in normal skin contain v6 segment. Thus, targeting v6 with antibody should have resulted in elimination of several CD44 proteins containing not only v6 but also other exons possibly required for normal skin barrier function. On the basis of our data, one may easily predict serious problems related to the proposed therapy. Also, identification of modified expression of a single exon is not sufficient to infer its pathogenicity, particularly CD44 fragments, which are not involved in attachment of the GAG side-chains. Instead, qualitative and quantitative combinations of various CD44 proteins seem to be related to particular functional states of epidermal cells.

Previous studies by immunohistochemistry and RT-PCR suggested that some specific isoforms of CD44 correlate with clinicopathological data and are involved in tumorigenesis of skin [42,43]. In our study, we have defined the exact compositions of all CD44 exons in epidermal keratinocytes. Depending on antibody used for immunohistochemistry for CD44, different outcomes could be obtained due to steric hindrance as a result of heavy glycosylation of the some exons of CD44. Our data provide a framework for understanding the full nature of all CD44 variants in human skin.

Recently, a comparative expression analysis of CD44 and ALDH1A1 putative cancer stem cell markers in various skin cancer subtypes was performed [41]. It was concluded that both melanoma and SCC show CD44-high phenotype suggesting that CD44 is a candidate for targeted therapy of skin cancers aiming at cancer stem cells. Since we have determined all CD44 transcripts, it would be interesting to determine which of the many *CD44* transcripts have a direct role in cancer cell stemness.

In conclusion, we determined full-length sequences of the 18 *CD44* transcripts in human epidermis using a unique cloning strategy and analysis of PCR products by PAGE. We showed that expression of a number of *CD44* transcripts was regulated by calcium, EGF, FCS, PMA, RA and hydrogen peroxide. Moreover, we found that normal and malignant keratinocytes produce different *CD44* transcripts in response to calcium and FCS, suggesting that specific CD44 isoforms are involved in tumorigenesis and differentiation via different CD44-mediated biological pathways. Our study should facilitate further analysis of CD44 functions in the skin, as well as in other tissues and cell lines

## Supporting Information

S1 Fig Nomenclature and examples of some *CD44* transcripts in humans. Filled boxes represent standard exons and empty boxes represent alternatively spliced exons. STD = standard.
(PDF)
