# Supplementary material for: Isolation of All CD44 Transcripts in Human Epidermis and Regulation of Their Expression by Various Agents
Source: PLoS One. 2016 Aug 9;11(8):e0160952. doi: 10.1371/journal.pone.0160952 (PMC4978388; doi:10.1371/journal.pone.0160952)
Supplement: S1 Fig — Filled boxes represent standard exons and empty boxes represent alternatively spliced exons. STD = standard. (PDF) [file pone.0160952.s001.pdf]

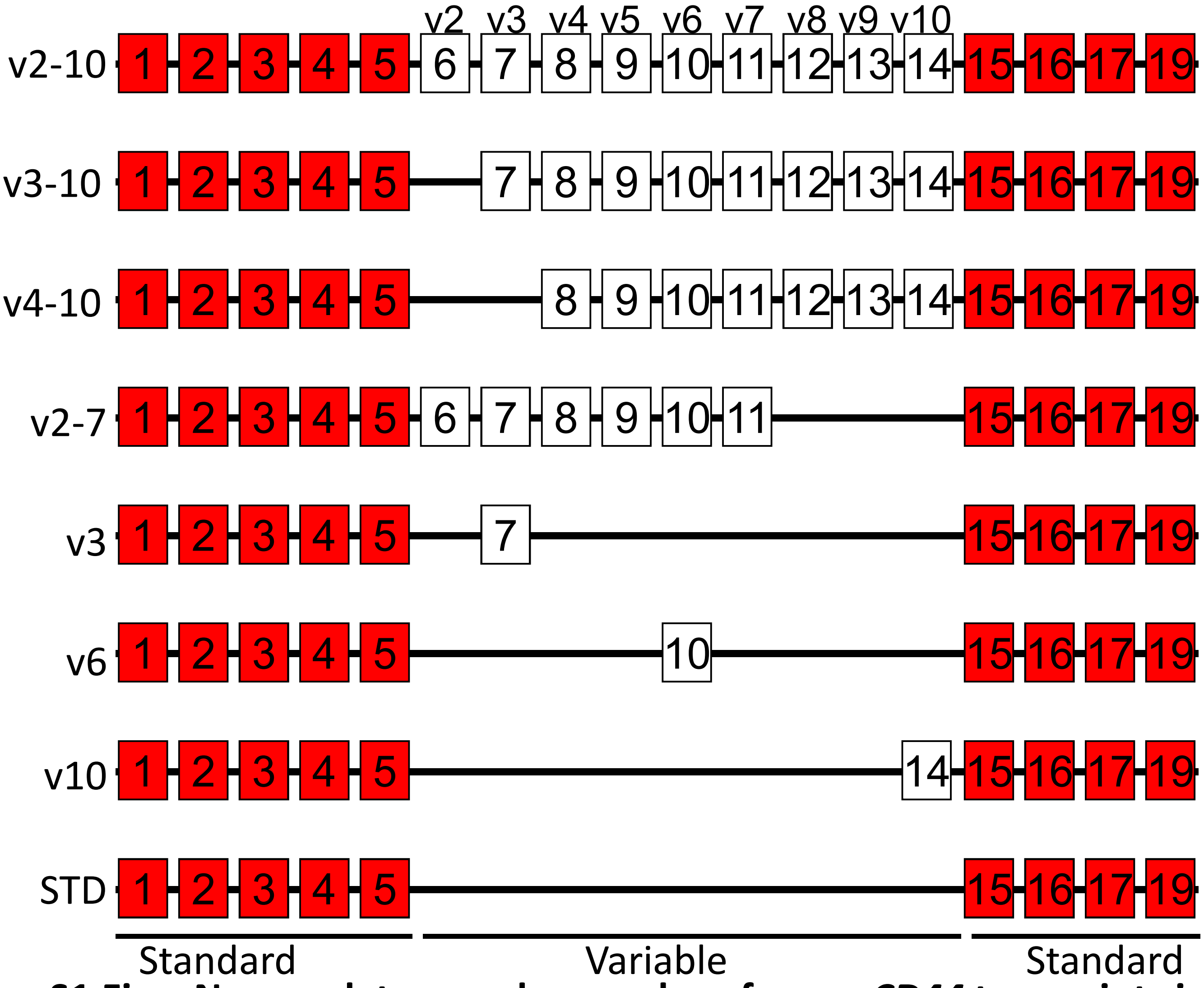

S1 Fig. Nomenclature and examples of some *CD44* transcripts in humans. Filled boxes represent standard exons and empty boxes represent alternatively spliced exons. STD=standard.
